# Automatic Assessment of ASPECTS Using Diffusion-Weighted Imaging in Acute Ischemic Stroke Using Recurrent Residual Convolutional Neural Network

**DOI:** 10.3390/diagnostics10100803

**Published:** 2020-10-09

**Authors:** Luu-Ngoc Do, Byung Hyun Baek, Seul Kee Kim, Hyung-Jeong Yang, Ilwoo Park, Woong Yoon

**Affiliations:** 1 Department of Radiology, Chonnam National University, Gwangju 61469, Korea; doluungoc@gmail.com (L.-N.D.); qorqod@hanmail.net (B.H.B.); kimsk.rad@gmail.com (S.K.K.); radyoon@jnu.ac.kr (W.Y.); 2 Department of Radiology, Chonnam National University Hospital, Gwangju 61469, Korea; 3 Department of Radiology, Chonnam National University Hwasun Hospital, Hwasun 58128, Korea; 4 Department of Electronics and Computer Engineering, Chonnam National University, Gwangju 61186, Korea; 5 Department of Artificial Intelligence Convergence, Chonnam National University, Gwangju 61186, Korea

**Keywords:** deep learning, diffusion magnetic resonance imaging, stroke

## Abstract

The early detection and rapid quantification of acute ischemic lesions play pivotal roles in stroke management. We developed a deep learning algorithm for the automatic binary classification of the Alberta Stroke Program Early Computed Tomographic Score (ASPECTS) using diffusion-weighted imaging (DWI) in acute stroke patients. Three hundred and ninety DWI datasets with acute anterior circulation stroke were included. A classifier algorithm utilizing a recurrent residual convolutional neural network (RRCNN) was developed for classification between low (1–6) and high (7–10) DWI-ASPECTS groups. The model performance was compared with a pre-trained VGG16, Inception V3, and a 3D convolutional neural network (3DCNN). The proposed RRCNN model demonstrated higher performance than the pre-trained models and 3DCNN with an accuracy of 87.3%, AUC of 0.941, and F1-score of 0.888 for classification between the low and high DWI-ASPECTS groups. These results suggest that the deep learning algorithm developed in this study can provide a rapid assessment of DWI-ASPECTS and may serve as an ancillary tool that can assist physicians in making urgent clinical decisions.

## 1. Introduction

Acute ischemic stroke is a major cause of disability, and urgent decision making for proper treatment strategies is critical for the improved outcome of patients with stroke [1,2]. The evaluation of infarct volume and stroke onset time play important roles in the early treatment approach [2]; hence the early detection and rapid quantification of the acute ischemic lesion on brain imaging with computerized tomography (CT) or diffusion-weighted magnetic resonance imaging (DWI) have become important for the diagnosis and treatment of acute ischemic stroke. 

The Alberta Stroke Program Early CT Score (ASPECTS) is an established 10-point semi-quantitative scoring system using brain CT and has been used for the rapid assessment of the extent of early ischemic changes in patients with acute anterior circulation stroke. Although the ASPECTS system has been widely utilized to determine the eligibility criteria of mechanical thrombectomy [3,4], the lack of agreement and the variability of ASPECTS among even experienced clinicians have been the main source of its limitation [5]. The DWI-ASPECTS, which is based on an ASPECTS measurement using DWI instead of CT, has been suggested as an alternative and shown to provide a superior inter-rater agreement and output prediction compared to CT-ASPECTS [6,7].

Recently, deep learning has been shown to be a powerful tool in computer vision, voice recognition, and natural-language processing, and has gained widespread attention for application in medical research. Convolutional neural networks (CNNs), a class of deep learning methods that has emerged as one of the most effective tools for pattern recognition, has been applied to the analysis of medical imaging, such as disease classification, lesion detection, segmentation, and data processing [8,9,10]. Although several recent studies have utilized deep learning algorithms to apply to CT and magnetic resonance imaging (MRI) data from stroke patients [11,12], the automated assessment of the extent of acute cerebral ischemia is still a challenging endeavor.

Building on previous work in which a 3D convolutional neural network (3DCNN) was applied for the binary assessment of DWI-ASPECTS [13], we developed a deep learning algorithm utilizing a recurrent residual convolutional neural network (RRCNN) for the automatic binary classification of DWI-ASPECTS from patients with acute anterior circulation ischemic stroke. We showed that the rapid assessment of DWI-ASPECTS using our proposed algorithm may provide a useful tool for physicians for categorizing these patients, which is of importance for time-sensitive clinical decision making.

## 2. Materials and Methods

### 2.1. Subjects

This retrospective study was approved by the Institutional Review Board of Chonnam National University Hospital, which waived the requirement for obtaining written consent. The data collection and all methods were carried out in accordance with the relevant guidelines and regulations. A total of 319 DWI datasets were included from patients who presented with acute anterior circulation stroke due to large vessel occlusion within 6 h of symptom onset at a tertiary stroke center from December 2010 and January 2016. An additional DWI dataset consisting of a total of 71 patients from February 2016 to November 2016 were collected for an independent test set. All patients underwent MRI examination using a 1.5T MRI scanner (Signa HDxt; GE Healthcare, Milwaukee, Wisconsin). DWI sequences were obtained in the axial plane by using a single-shot, spine-echo echoplanar technique with the following parameters: repetition time of 9000 ms, echo time of 80 ms, slice thickness of 4 mm, intersection gap of 0 mm, field of view (FOV) of 260 × 260 mm, matrix size of 128 × 128 (approximately 2 mm × 2 mm in-plane resolution) and b-values of 0 and 1000 s/mm^2^. 

**Figure 1:**
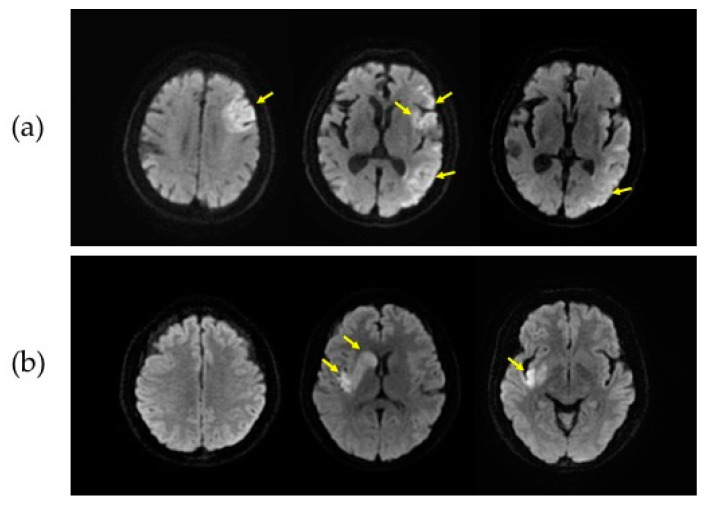
Representative diffusion-weighted magnetic resonance imaging (DWI) slices from two groups. DWI data from a patient with the Alberta Stroke Program Early Computed Tomographic Score (ASPECTS) of 5 (**a**) and the ASPECTS of 7 (**b**) are shown. The slices in the first, second and third column in each patient correspond to the supraganglionic, ganglionic, and infraganglionic levels, respectively. The yellow arrows indicate infarct lesions.

DWI-ASPECTS was retrospectively assessed by two neuroradiologists who were blinded to clinical information. The assessment of DWI-ASPECTS involves 10 distinct regions, subdividing the territory of the middle cerebral artery, in which the overall score is determined by deducting a score of 1 from the initial score of 10 for each affected region [14]. Approximately 95% of the DWI-ASPECTS assessment (370 out of 390 cases) were the same between the two neuroradiologists. The final conclusions for the disagreeing 20 cases were made by a consensus from the two neuroradiologists after a further inspection by them. Patients were classified into two groups according to their DWI-ASPECTS: group 1 consisted of patients with the DWI-ASPECTS of 1–6 (*n* = 147) and group 2 with the DWI-ASPECTS of 7–10 (*n* = 243). This binary classification was based on the previous finding, which demonstrated the distinct clinical outcome between the two groups and consequently the need for rapid determination of differential treatment options between these two groups [15,16]. Figure 1 shows an example of DWI images from the two groups.

Eighty percent of the 319 DWI data points were randomly selected for the training dataset (*n* = 244) and the remaining 20% were kept for validation (*n* = 75). The additional dataset (*n* = 71) was used for the independent testing. The distribution of the ASPECTS among the training, validation, and independent test set is shown in Table 1.

### 2.2. Slices Filtering

A slice filtering strategy was applied to bypass DWI slices that were non-informative. Each DWI dataset contained a sequence of approximately 40 imaging slices. Approximately 15% of cranial and 35% of caudal slices of each patient data, which were well outside the area of the middle cerebral arterial territory and uninformative for estimating ASPECTS, were removed. As a result, approximately 20 slices, with regions that included the middle cerebral arterial territory, remained.

### 2.3. DWI Preprocessing

**Figure 2:**
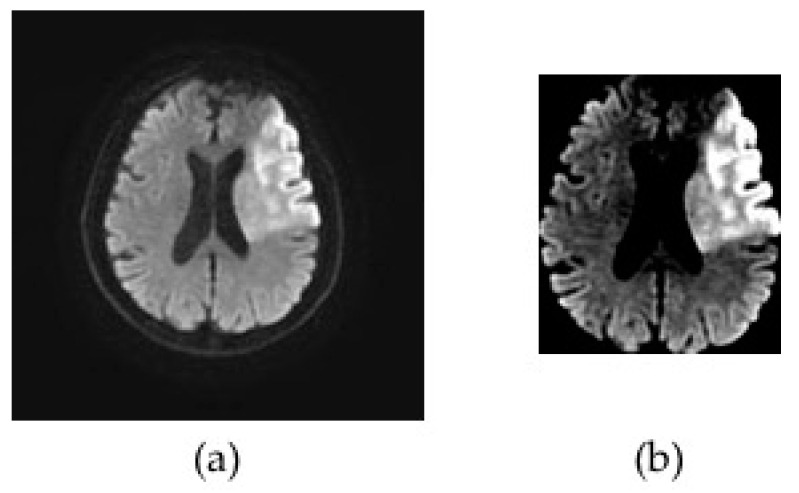
Representative diffusion-weighted magnetic resonance imaging (DWI) images illustrating the preprocessing step. The original DWI image (**a**) was cropped around brain parenchyma and a contrast stretching algorithm was applied to enhance the contrast of the DWI data (**b**).

After the slice filtering, the DWI datasets were preprocessed by brain cropping and a contrast stretching (Figure 2). The brain cropping was intended to remove the background portion of the images and to include only brain parenchyma. Based on the pixel intensity values from the image histogram, the top, bottom, left and right boundary pixels of the brain parenchyma were determined and utilized for selecting the boundary for cropping. The contrast of the cropped image was enhanced by the contrast stretching algorithm using the following formula:

$$P_{out}=\left(P_{in}-P_{in}^{min}\right)\left(\frac{P_{out}^{max}-P_{out}^{min}}{P_{in}^{max}-P_{in}^{min}}\right)+P_{out}^{min}$$

where *P_out_* is the pixel value of the contrast-stretched image and *P_in_* is the pixel value of the original image. $P_{out}^{max}$ and $P_{out}^{min}$ are 255 and 0, respectively. $P_{in}^{max}$ and $P_{in}^{min}$ are the 99th and 80th percentile of the histogram in the original image, respectively.

### 2.4. Data Augmentation

**Figure 3:**
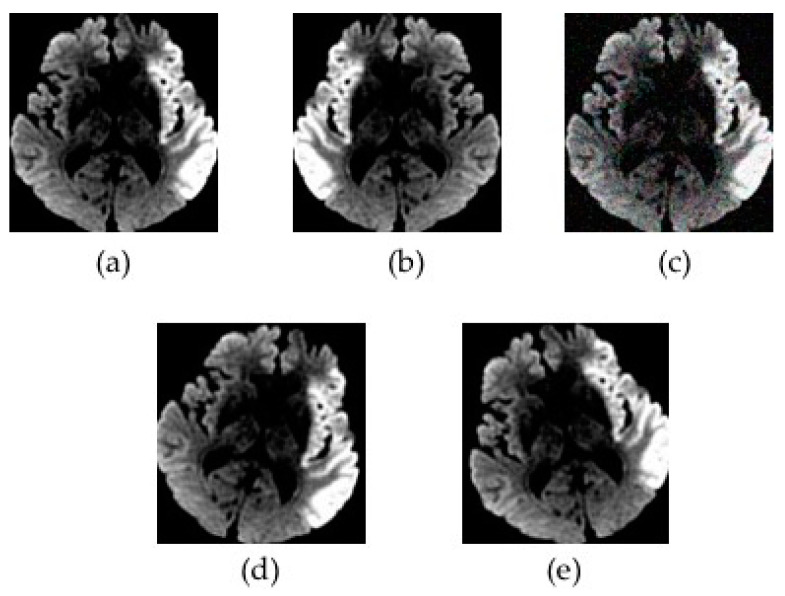
An illustration of data augmentation. The preprocessed diffusion-weighted magnetic resonance imaging data (**a**) underwent a horizontal flip (**b**), the addition of Gaussian noise (**c**), a clockwise 15-degree rotation (**d**), and a counter-clockwise 15-degree rotation (**e**).

Every 2D imaging slice of the DWI datasets was resized to 80 × 80 by a bicubic interpolation, rendering a final input DWI sequence with a 20 × 80 × 80 resolution. The DWI datasets used for training the model were augmented by a horizontal flip, the addition of Gaussian noise, and a clockwise and counter-clockwise 15-degree rotation as shown in Figure 3. After the data augmentation, the training set consisted of 1220 DWI samples, corresponding to a total of 24,400 imaging slices.

### 2.5. Model Training

**Figure 4:**
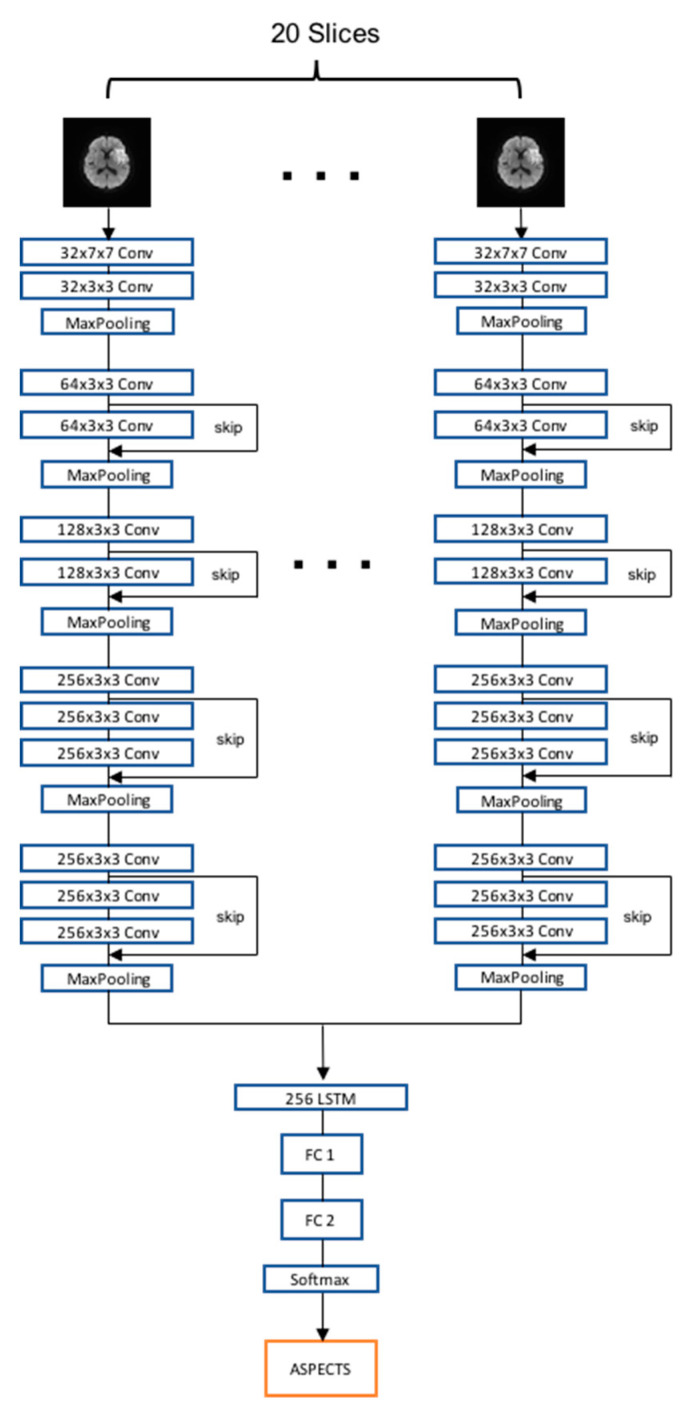
The flowchart of the recurrent residual convolutional neural network model developed in this study. The proposed model contained five convolution blocks followed by a recurrent block, fully connected layers, and a softmax classifier. LSTM, long short term memory; FC, fully connected layer; ASPECTS, Alberta Stroke Program Early Computed Tomographic Scoring.

We used the RRCNN for model training, which considered the DWI slices as a sequence of images. It contained five convolution blocks for extracting 2D features and one recurrent block for extracting sequential features. The proposed RRCNN structure was adapted from the VGG16 [17] and ResNet [18] structure by adding skip connections in each convolution block as shown in Figure 4. Each block of convolution had two or three convolution layers with the kernel size of 3 × 3 or 7 × 7 and one max pooling layer. The number of feature maps in each convolution block was 32, 64, 128, 256, and 256, respectively. The recurrent block contained one long short term memory (LSTM) layer with 256 hidden nodes. The DWI datasets underwent a slice filtering, preprocessing, and augmentation step before training. We used an Adam optimizer with a learning rate of 1e-6 and a batch size of 32. Our models were trained using Tensorflow-GPU with NVIDIA GTX 1080 Ti.

### 2.6. Comparison of RRCNN with Pre-Trained Models and 3DCNN

We evaluated and compared the performance of the proposed RRCNN structure with those of VGG16 [17] and Inception V3 [19] models that were pre-trained using ImageNet data [20]. The pre-trained models were fine-tuned by adding one LSTM layer before the fully connected layers. During training, the weights on the LSTM layer and the fully connected layers were updated, while the pre-trained CNN weights were frozen. We used an Adam optimizer with a learning rate of 1e-5 and a batch size of 32. In addition, we trained the 3DCNN model [21], which considered the DWI slices as three-dimensional data, and compared its performance with that of the proposed RRCNN model. The learning rate and the batch size of 3DCNN was 1e-5 and 32, respectively. Detailed information about the 3DCNN model has been previously described [13]. All deep learning models were trained and evaluated three times using the validation set in order to assess and verify the performance of their training and the mean performances were reported. Sensitivity (Group 1 considered as a positive condition), specificity, F-score, accuracy with a threshold of 0.5 and the area under the curve (AUC) from the receiver operating characteristic (ROC) curve was calculated.

## 3. Results

The ability of our proposed RRCNN for the classification of DWI data between patients with a low and high DWI-ASPECTS was demonstrated and compared with other deep learning algorithms. Table 2 shows the comparison of results between the proposed RRCNN, the pre-trained VGG16 and Inception V3 model, and the 3DCNN. For the validation data, the accuracy of the proposed RRCNN was 84.4%, which was higher than those of the pre-trained VGG16 (72.8%), pre-trained Inception V3 (72.4%), and 3DCNN (81.7%). Similarly, the AUC of the proposed RRCNN was 0.910, which was higher than those of the pre-trained VGG16 (0.801), pre-trained Inception V3 (0.834), and 3DCNN (0.844). The sensitivity and F1 score also showed a similar trend. The specificity of RRCNN and 3DCNN were similar (89.8% and 89.1%, respectively), which were slightly higher than those of pre-trained VGG16 (86.3%) and pre-trained Inception V3 (84.3%).

Using the independent test dataset, the proposed RRCNN model demonstrated a reasonable performance with a sensitivity, specificity, F1-score, accuracy, and AUC of 83.9%, 90.0%, 0.888, 87.3%, and 0.941, respectively. For all CNN models, the specificity, F1 score, accuracy and AUC of the independent test were either slightly higher than or comparable to those of the validation test. The sensitivities of the RRCNN and 3DCNN were comparable between the validation and independent test datasets, while the sensitivities of pre-trained models in the validation set were higher than those in the independent set. In general, the evaluation metrics showed that the proposed RRCNN had comparable performances with slightly higher levels of sensitivity, specificity, F1 score, accuracy and AUC compared to the other three models. The high level of F1 score (0.888) and the relatively small difference between the sensitivity (83.9%) and the specificity (90.0%) of the RRCNN model indicate that our proposed model did not have a critical bias toward a specific ASPECTS class. 

**Figure 5:**
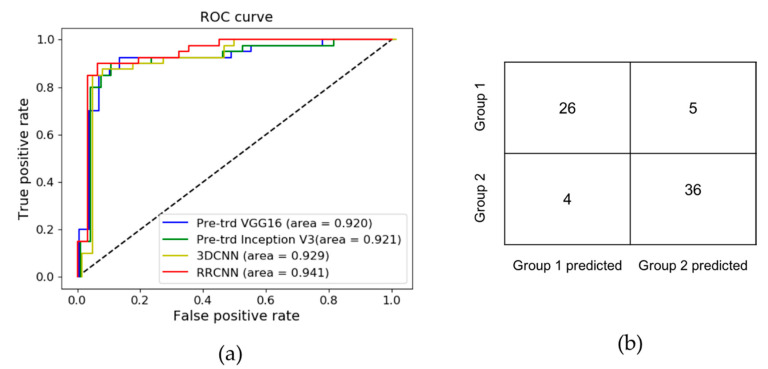
Summary of results using the independent test set. The receiver operating characteristic (ROC) curves are shown for the pre-trained VGG16, pre-trained Inception V3, 3D convolutional neural network (3DCNN), and proposed recurrent residual convolutional neural network (RRCNN) models (**a**). The proposed RRCNN model demonstrated a slightly higher area under the curve (AUC) (0.941) compared to other models. The confusion matrix demonstrates the classification results using the independent test set (**b**).

Figure 5a shows the comparison of ROC curves between the four deep learning models. The confusion matrix of the RRCNN model on the independent test data is shown in Figure 5b. Among five cases that were incorrectly predicted as Group 2, three belonged to ASPECTS 6, one ASPECTS 5, and one ASPECTS 4. Among four cases that were incorrectly predicted as Group 1, two belonged to ASPECTS 7, and the other two to ASPECTS 8.

## 4. Discussion

Estimating the extent of infarction volume plays a pivotal role in the management of patients with acute ischemic stroke and has shown to be an important factor in determining the eligibility of reperfusion therapy and predicting clinical outcomes [4,6,7]. CT-based ASPECTS as well as DWI-ASPECTS are widely used tools for the indirect and rapid assessment of infarction volume [3,5,6,7]. Several efforts have been made to automate the process of estimating the extent of infarction using computer software. e-ASPECTS is one such method, which was proposed by Hampton-Till et al. and designed for the automated scoring of CT-based ASPECTS [14]. Several studies have demonstrated the feasibility of using e-ASPECTS in assessing CT scans of acute ischemic stroke patients and suggested that the software achieved a performance comparable to stroke physicians or neuroradiologists [22,23]. Other studies have shown that the software can be used to predict the outcome after mechanical thrombectomy [24,25].

Although CT scan is commonly used for the assessment of infarct lesion for stroke patients because of its availability in most emergency clinical environments, the MRI-based assessment of infarction, including DWI-ASPECTS, has shown to provide alternative methods [15,16,26,27]. In a recent study, automated computer-based ASPECT scoring was applied to DWI [28]. The authors utilized a decision tree algorithm to develop an automated method to predict the total ASPECT score. Although the performance of their automated method was slightly worse than human expert scoring, they demonstrated that the machine learning-based method can be used to determine DWI-ASPECTS with good precision. Another automated ASPECTS system combining feature engineering and random forest learning was developed with non-contrast CT scans of 257 patients with acute ischemic stroke using DWI as the ground truth and demonstrated the ability to determine ASPECTS using an automated approach [29]. These studies suggest that machine learning algorithms can be utilized for the automated assessment of ASPECTS.

Deep learning algorithms have been applied to medical imaging [9] and have led to an exciting opportunity for data-driven stroke management and guiding the diagnosis of acute ischemic stroke [30,31]. Recently, several studies have used CNN algorithms for application in acute ischemic lesions [32,33,34,35,36] and provided effective tools for automatic lesion segmentation or volume calculation. Other studies have focused on developing a deep learning-based approach for detection or identification of large vessel occlusion from CT angiography [37,38]. These studies suggest that deep learning algorithms can be effectively applied to the management of stroke.

Although previous efforts using e-ASPECTS have been shown to provide quantitative scores of ASPECTS, we focused on developing a deep learning algorithm for a fast rendering of a binary classification of DWI-ASPECTS. In ischemic stroke, time is brain, meaning delays to proper treatment lead to worse brain injuries and damage [1,2]. Currently, endovascular treatment is regarded as the standard treatment for selected acute ischemic stroke patients due to large vessel occlusion; however, an appropriate patient selection based on rapid decision-making is important for producing maximal clinical outcome [39]. Previous findings reported marked differences in clinical outcome between two subgroups. The patients with DWI-ASPECTS greater than or equal to 7 had a distinct clinical outcome compared to those with DWI-ASPECTS smaller than 7 after intra-arterial or IV pharmacologic thrombolysis [15,16]. These findings signified the need for determining the different treatment strategies based on the rapid characterization of ischemic lesions between the two groups. The results from our findings suggest that the RRCNN model developed in this study may provide an important ancillary tool for clinicians in a time-sensitive assessment of DWI-ASPECTS from acute ischemic stroke patients. With further improvements and clinical validations, we envision that the algorithm developed in this study may be used for building a triage and fast response system where it can provide a shorter time to mechanical thrombectomy and also a faster time to transfer from a peripheral hospital to a tertiary stroke center, so that the relevant procedure can be performed. This would present a significant clinical benefit because there is a shortage of interventional neuroradiologists [40] and the automated DWI-ASPECTS assessment can help accelerate the procedure of identifying patients who need an interventional neuroradiologist’s consultation. Most recently, Viz LVO (Viz.ai, Inc. San Francisco CA, USA), which is software with the similar aim to improve the triage and shorten the time-to-treatment for stroke patients using CT angiography, became the first artificial intelligence-based software to receive the Medicare New Technology Add-on Payment (NTAP) by the Centers for Medicare & Medicaid Services [41,42].

In our previous effort, we provided a similar approach for the rapid assessment of DWI-ASPECTS using a 3DCNN model. Although the previous results demonstrated an accuracy of 81% and AUC of 0.872 for the binary classification DWI-ASPECTS, the 3DCNN possessed more than 100 million parameters, requiring a substantial amount of computation for training. The current study presents several advancements over the previous one. A larger number of data were included in the current study (390 DWI data in the current study vs. 308 in the previous study). In this study, we developed our proposed model based on a recurrent neural network (RNN), which has exhibited promising results in recognizing patterns in sequences of data [43]. In this setting, the multi-slice DWI datasets were regarded as a sequence of images, rather than 3D images. The performance of the proposed RRCNN model was compared to that of the 3DCNN model that has been extensively used for processing 3D datasets [21]. Although the results from the two models were comparable, with our RRCNN model showing a slightly higher level of accuracy (87.3%) and AUC (0.941) compared to the 3DCNN model (accuracy, 84.5%; AUC, 0.929), the computation cost of the proposed RRCNN model was notably reduced compared to that of the 3DCNN, requiring approximately 4 to 5 h of training time, while the 3DCNN required more than 10 to 12 h of training time. These results suggest that RNN-based models may provide an alternative way to analyze multi-slice medical imaging data.

VGG16 [17] and Inception V3 [19] are two of the popular deep neural networks that have been shown to be very efficient in image classification. Although these networks, which were pre-trained with large-scale data from ImageNet [20], have been widely applied to medical image analysis in combination with transfer learning and fine tuning technique [44,45,46,47,48], the effectiveness of the transfer learning method using these deep networks is debatable [49] because medical images, such as MRI and CT, are very different from the images in ImageNet, which are mostly natural images, and the high-level features that are learned during training of medical images can be very different. In addition, the application of transfer learning using these deep networks may need careful consideration depending on the training condition, such as the number of training datasets, because these complex neural networks generally require a large amount of training data to be effectively trained. Nevertheless, pre-training the well-known networks and applying the transfer learning technique are still very popular methods for medical image analysis. We compared the performance of the proposed RRCNN model that was trained from scratch to those of pre-trained VGG16 and Inception V3. Although this comparison may provide limited information given the data and the specific classification task we applied, the results may be used as a reference regarding the choice of CNN model and training strategy for future studies. The results from our study suggest that the proposed RRCNN model, which has a smaller number of layers and parameters compared to VGG16, Inception V3 and 3DCNN, proved to be as effective, if not more effective, for a rapid assessment of ASPECTS with a limited number of training datasets (Table 2). A recent study reported a similar observation that the pre-training method using images from ImageNet sped up convergence early in training, but did not necessarily provide improved regularization, nor increase test accuracy [49,50].

Although our study demonstrated the potential of the deep learning model to be used for the rapid, automatic assessment of early acute ischemic changes in DWI, it still presents several limitations. First, our study aimed at differentiating low ASPECTS (DWI-ASPECTS 1–6) from high ASPECTS (DWI-ASPECTS 7–10) groups, thereby presenting a global estimation of DWI-ASPECTS rather than a classification of individual DWI-ASPECTS regions. The multi-class classification of individual ASPECTS or a region-based approach may provide an added prognostic value and will be considered in future research. Second, our study included a total of 319 patient DWI datasets acquired from an MR scanner located in our emergency department. Although our results demonstrated the feasibility of using deep learning models for the automatic classification of DWI-ASPECTS, the collection of a larger number of datasets should be considered to improve the performance of the model. In addition, the inclusion of data acquired from MR scanners of different vendors needs to be considered in order to improve the general applicability of our results. A concerted effort to collect data from multiple institutions and validate the developed model with different external datasets is on-going, which is expected to contribute not only to further validate our model, but also to accumulate and share MRI data from stroke patients that can be used in future research. The additional datasets that will be available from the coordinated multi-center collaboration may allow us to develop deep learning models that are able to perform a multi-class classification of DWI-ASPECTS or a region-based analysis of ASPECTS.

## 5. Conclusions

We developed a deep learning algorithm based on a recurrent residual convolutional neural network for the classification of DWI-ASPECTS. Our model demonstrated an accuracy of 87.3% and AUC of 0.941 for automatic classification between the low and high DWI-ASPECTS. The results suggest that the deep learning algorithm developed in this study can serve as an ancillary tool that assists in the rapid decision making for patients with acute ischemic stroke.

## Figures and Tables

**Table 1:** Distribution of the Alberta Stroke Program Early Computed Tomographic Score (ASPECTS) among the training, validation, and independent test set.

	ASPECTS										Group 1 (ASPECTS 1–6)	Group 2 (ASPECTS 7–10)	Total
	1	2	3	4	5	6	7	8	9	10			
Training	1	4	5	13	35	32	48	65	39	2	90	154	244
Validation	0	0	2	3	12	9	14	20	14	1	26	49	75
Testing	0	3	4	7	5	12	9	19	9	3	31	40	71

**Table 2:** Comparison of model performances between various deep learning algorithms for classifying patients with high diffusion-weighted magnetic resonance imaging-Alberta Stroke Program Early Computed Tomographic Score (DWI-ASPECTS) (7–10) and low DWI-ASPECTS (1–6).

Model	Validation Dataset					Independent Test Dataset				
	Sens. ^4^ (%)	Spec. ^5^ (%)	F1	Acc. ^6^ (%)	AUC	Sens. ^4^ (%)	Spec. ^5^ (%)	F1	Acc. ^6^ (%)	AUC
Pre-trd. ^1^ VGG16	70.5	86.3	0.801	72.8	0.801	61.3	92.5	0.831	78.8	0.920
Pre-trd. ^1^ Inception V3	71.8	84.3	0.795	72.4	0.834	64.5	92.5	0.840	80.0	0.921
3DCNN^2^	78.2	89.1	0.848	81.7	0.844	77.4	90.0	0.867	84.5	0.929
Proposed RRCNN^3^	82.0	89.8	0.872	84.4	0.910	83.9	90.0	0.888	87.3	0.941

^1^ Pre-trd., Pre-trained; ^2^ 3DCNN, 3D convolutional neural network; ^3^ RRCNN, recurrent residual convolutional neural network; ^4^ Sens., Sensitivity; ^5^ Spec., Specificity; ^6^ Acc., Accuracy
